# Crosswalk and correlation between the Oxford Knee Score (OKS), Knee Society Score (KSS) and Knee Injury and Osteoarthritis Outcome Score (KOOS-12) in knee osteoarthritis

**DOI:** 10.1186/s41687-026-01117-x

**Published:** 2026-06-29

**Authors:** Shaun Kai Kiat Chua, Linus Ren Hao Tan, Chien Joo Lim, Michelle Jessica Pereira, Bryan Yijia Tan

**Affiliations:** 1 Department of Orthopaedic Surgery, Woodlands Hospital, National Healthcare Group, 17 Woodlands Dr 17, Singapore, 737628 Singapore; 2 Physiotherapy Department, Sengkang General Hospital, 110 Sengkang East Way, Singapore, 544886 Singapore; 3 Health Services and Outcomes Research, National Healthcare Group, National Skin Centre, 1 Mandalay Road, Level 4, Singapore, 308205 Singapore

**Keywords:** Knee, Osteoarthritis, Correlation, Outcome measures, Crosswalk, PROM, KOOS-12, OKS, KSS

## Abstract

**Background:**

Significant heterogeneity exists in the patient-reported outcome measures (PROMs) used to assess outcomes in knee osteoarthritis (KOA), with limited evidence exploring the correlation and conversion among the various PROMs. Our study aims to explore the correlation and conversion between the Oxford Knee Score (OKS), Knee Injury and Osteoarthritis Outcome Score (KOOS-12) and Knee Society Score (KSS) in patients with non-operatively treated KOA.

**Methodology:**

This was a prospective cohort study of 131 patients who received non-operative treatment (standardized physical therapy and analgesia) for KOA between July 2020 and January 2024. Three validated PROMs for KOA (OKS, KOOS-12 and KSS) were measured for patients at baseline, 3-months, 6-months, 1-year and 2-years follow-up. Overall, 498 complete paired outcome scores (KSS/OKS/KOOS-12 at a single time point) were included for analysis. The data were then randomly split into a derivation (75%) and internal validation (25%) cohort. The correlation between the outcome scores were calculated, and linear regression model was used to obtain conversion equations between the measured outcomes. Internal validation of the derived conversion equation was performed subsequently by comparing the mean difference between the equation-derived and actual scores.

**Results:**

A significant strong positive correlation was found between KOOS-12 and OKS (coefficient:0.79;*p* < 0.001), and a significant moderate positive correlation was found between the KOOS-12 and KSS (coefficient:0.50;*p* < 0.001). A significant moderate positive correlation was found between the OKS and KSS (coefficient:0.67;*p* < 0.001). Six conversion equations derived for the measured PROMs had low mean difference between the equation-derived and actual scores (0.31–1.68). Moderate to strong correlation was found between the actual and equation-derived scores, with weakest strength observed in the conversion between KOOS-12 and KSS (coefficient:0.53) and the strongest strength observed in the conversion between KOOS-12 and OKS (coefficient:0.80).

**Conclusions:**

On internal validation, we found good correlation between the mean actual and equation-derived outcome scores despite significant within-individual variability. The conversion equations derived in this study can facilitate pooling and comparison of studies using heterogenous outcomes measures and meta-analysis to increase statistical power and address novel questions in KOA. Nonetheless, the equations should not be utilized to predict individual scores from one to another due to high within-individual variability.

**Clinical trial number:**

Not applicable.

## Background

Over the years, a myriad of scoring systems has been developed in orthopaedic practice to measure patient outcomes over time to guide treatment decisions. Given the diversity of outcome measures available, harmonising and mapping scores between instruments is essential to ensure comparability across studies and populations [1]. 

For knee osteoarthritis (KOA), various clinician-measured and patient-reported outcome measures (PROMs) have been used to assess the severity of symptoms and functional status of affected patients [2]. Among a growing number of scoring systems used to measure outcomes in patients with knee pathologies, the Knee Society Score (KSS), Oxford Knee Score (OKS) and Knee Injury and Osteoarthritis Outcome Score-12 (KOOS-12) are widely employed validated measures to evaluate clinical outcomes in patients with KOA [2–5]. The OKS is a 12-item questionnaire measuring pain and function that was originally developed to assess outcomes following total knee arthroplasty, although it has since been validated for use across various stages of knee OA [5, 6]. In contrast, the KOOS-12 is a short-form version of the Knee Injury and Osteoarthritis Outcome Score (KOOS), focusing on three domains: pain, function in daily living, and knee-related quality of life [3]. KOOS-12 has been described to give a more comprehensive picture of the patient’s lived experience and it has also been validated as a reliable and responsive measure for assessing knee health in patients with osteoarthritis [7]. Finally, the KSS is another validated scoring system with a composite nature including both a subjective patient-reported function score component and an objective clinical knee score component, that is commonly used to assess outcomes in KOA patients before and after knee arthroplasty [4]. Despite their complementary roles, the differences in scaling and item content make it difficult to directly compare results or synthesize findings across studies that use different tools.

The concept of “crosswalks” or validated equations to convert scoring systems between one another have been applied in various domains of medicine and orthopaedic care, including a number of crosswalk studies conducted in the field of knee arthroplasty [8–11]. Soh et al. performed a crosswalk of KOOS-12 and OKS in patients undergoing knee arthroplasty while Bilbao et al. studied the relationship between Western Ontario and McMaster Universities Osteoarthritis Index (WOMAC) and EQ-5D-5 L in KOA [10, 12]. Nonetheless, to the authors’ knowledge, there remains no crosswalk studies on KSS, KOOS-12, and OKS in the context of non-operatively treated knee osteoarthritis patients. Understanding the relationships between these different outcome measures (OKS, KSS, KOOS-12) could provide a valuable tool for data harmonization across studies to facilitate comparison of these heterogenous outcome measures and meta-analysis in KOA. Furthermore, the development of crosswalks between instruments with overlapping constructs (e.g. OKS, KOOS-12, KSS for knee pain and function) can reduce responder burden by allowing clinicians to administer a simpler instrument (among highly correlated instruments) while still allowing conversion and comparison with historical or external data collected using the other instruments [8]. Therefore, our study aims to explore the correlation between OKS, KOOS-12, and KSS in non-operatively treated KOA, and formulate a set of regression equations that would allow the conversion and prediction of these scores from one another.

## Methods

### Study design

This was a multicenter prospective cohort study of general orthopedic patients with knee OA who received receiving standard non-operative treatment (standardized physical therapy, analgesia). Results were reported in alignment with the Strengthening the Reporting of Observational Studies in Epidemiology (STROBE) guidelines [13]. 

### Ethical approval

Ethical approval for this study was obtained from the National Healthcare Group Domain Specific Review Board (reference number WHC/2020-00076). Informed consent was obtained from all participants.

### Study population

This study included patients from two tertiary hospitals who received non-operative treatment of knee OA between July 2020 to January 2024. A total of 912 patients were screened, 556 rejected participation, 31 enrolled but terminated before data collection, OKS and KSS measures were not collected on 194 patients, leaving 131 patients included into the study. All patients were community ambulant independently (with or without any walking aids) and fulfilled the National Institute for Health Care Excellence (NICE) clinical diagnostic criteria for knee OA (i.e. 45 years of age or older, presence of activity related joint pain, and absence of morning stiffness that is joint related lasting more than 30 minutes) [14]. Patients with any previous history of knee surgery, secondary arthritis (e.g. inflammatory arthritis), presence of alternative diagnosis to account for their knee symptoms (e.g. referred pain), patients requiring wheelchair for mobilization or medical comorbidities severely limiting activities of daily living (ADL) such as previous strokes or chronic lung conditions with poor effort tolerance were excluded from the study. Patients were followed up for 2 years from their first consultation visit.

### Descriptive data

The baseline demographic characteristics (age, gender, ethnicity, body mass index (BMI), employment status) and disease characteristics (side of arthritis, disease severity – Kellgren and Lawrence (KL) grade, duration of OA) were collected for patients.

### Outcome measures

Three widely validated scoring systems (KSS, OKS, KOOS-12) were measured for patients at 5 different time points – at baseline, 3-months, 6-months, 1-year and 2-years follow up from the initial visit. The English versions of KSS, OKS, and KOOS-12 were used in this study. The KOOS-12 is a questionnaire measuring the patient’s personal perception of their knee function, pain, symptoms, and knee-related quality of life. It consists of 12 items covering 3 domains (each domain consisting of 4 items), namely pain, activities of daily living (ADL) and quality of life (QoL). Each item is given a score of 0 to 4, with higher scores representing superior outcomes [3]. The KSS is a scoring system for rating both the knee and patient’s functional abilities. It encompasses two main domains, namely the knee/ objective domain (alignment, knee pain, range of motion, knee stability) and function/ subjective domain (fulfilment of expectations, satisfaction with outcome, ability to perform functional activities). Each domain is scored between 0 and 100, with higher scores indicating a better knee condition [4]. Only the subjective (function) domain of KSS was assessed for this study. The OKS is a patient-reported questionnaire with consist of 12 items used to assess knee function and pain. Each item is given a score between 0 and 4 (total scores ranging from 0 to 48), with higher score indicating better knee function and lower pain levels [5]. 

### Crosswalks between KSS, OKS, and KOOS-12

Crosswalks are statistically derived equations that enables the translation of scores between different outcome measures intended to assess related constructs, thereby facilitating pooling, comparison, and synthesis of data across studies involving heterogenous instruments [15]. In practice, crosswalk equations may be applied at the individual or group level, depending on the degree of construct equivalence between measures, and strength of internal and external validation of the equations at the group and individual level respectively [8]. The statistical approach used to derive the crosswalks between KSS, OKS, and KOOS-12 in our study is detailed below.

### Statistical analysis

Descriptive statistics was used to describe the demographic characteristics of the participants. The outcome scores at different time points were presented as mean with standard deviation and median with interquartile range. A total of 498 paired observations for KOOS-12, OKS and KSS scores across all time points were collected and split into derivation cohort consisting of 75% of the samples (373 observations) to derive the conversion equation, and the equations was tested with remaining 25% of the samples (125 observations) as internal validation. The splitting of samples to derivation cohort and internal validation cohort was done through simple random sampling to ensure the two groups has similar characteristics.

For derivation cohort, scatter plots were created for each pairwise comparison as overall with correlation between the pairwise outcomes were tested using Pearson correlation. Six linear regression models were used to come out with the conversion equations between OKS, KSS and KOOS-12 (OKS to KOOS-12, KOOS-12 to OKS, OKS to KSS, KSS to OKS, KOOS-12 to KSS and KSS to KOOS-12), adjusting for age and gender. Multicollinearity was checked using Variance Inflation Factor (VIF), and assumptions of heteroskedasticity was checked using Bruesch-Pagan / Cook-Weisberg test.

Internal validation was performed using the validation cohort, and predicted scores were computed based on the equation derived from the derivation cohort to compare with the actual/observed scores. Mean difference with 95% confidence intervals and the range (minimum – maximum scores) of the difference between observed and predicted outcome scores was computed to explore the precision or error or margines of the converted scores. In addition, the correlation between actual and predicted scores was tested using Pearson correlation. Statistical significance was denoted by p< 0.05 and all the analyses were performed using STATA version 14.0.

## Results

### Patient baseline demographics

This study included 131 patients who received non-operative management of knee OA. The mean age was 63.1 years, with 66.4% (87/131) being females and majority being Chinese (77.9%, 102/131). Among the included patients, 18.7% (23/131) had a Kellgren and Lawrence (KL) grade 2 knee OA, while 45.5% (56/131) and 35.8% (44/131) had a KL grade 3 and KL grade 4 knee OA respectively. Further details of the patient’s baseline demographic characteristics can be found in Table 1.

### Measured outcomes

The outcome measures (OKS, KSS, KOOS-12) measured for the patients at the 5 different timepoints on follow up (baseline, 3-months, 6-months, 1 year, 2 year) are as summarized in Table 2. Overall, 498 complete paired outcome scores (KSS/OKS/KOOS-12 measured at a single time point) were included for analysis.

**Fig. 1:**
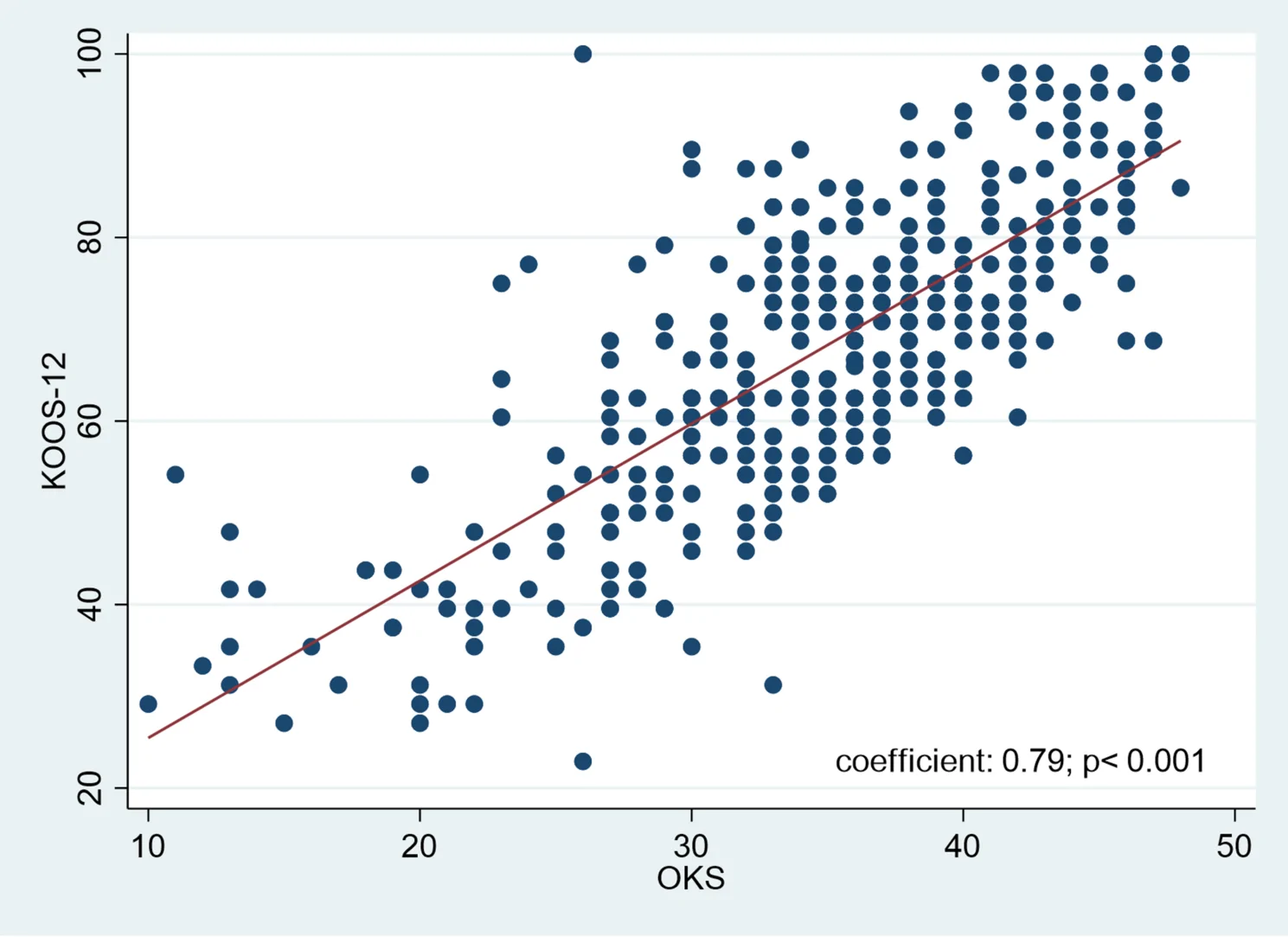
Correlation between OKS and KOOS-12. A significant positive, strong correlation was observed between KOOS-12 and OKS (coefficient: 0.79; p< 0.001)

**Fig. 2:**
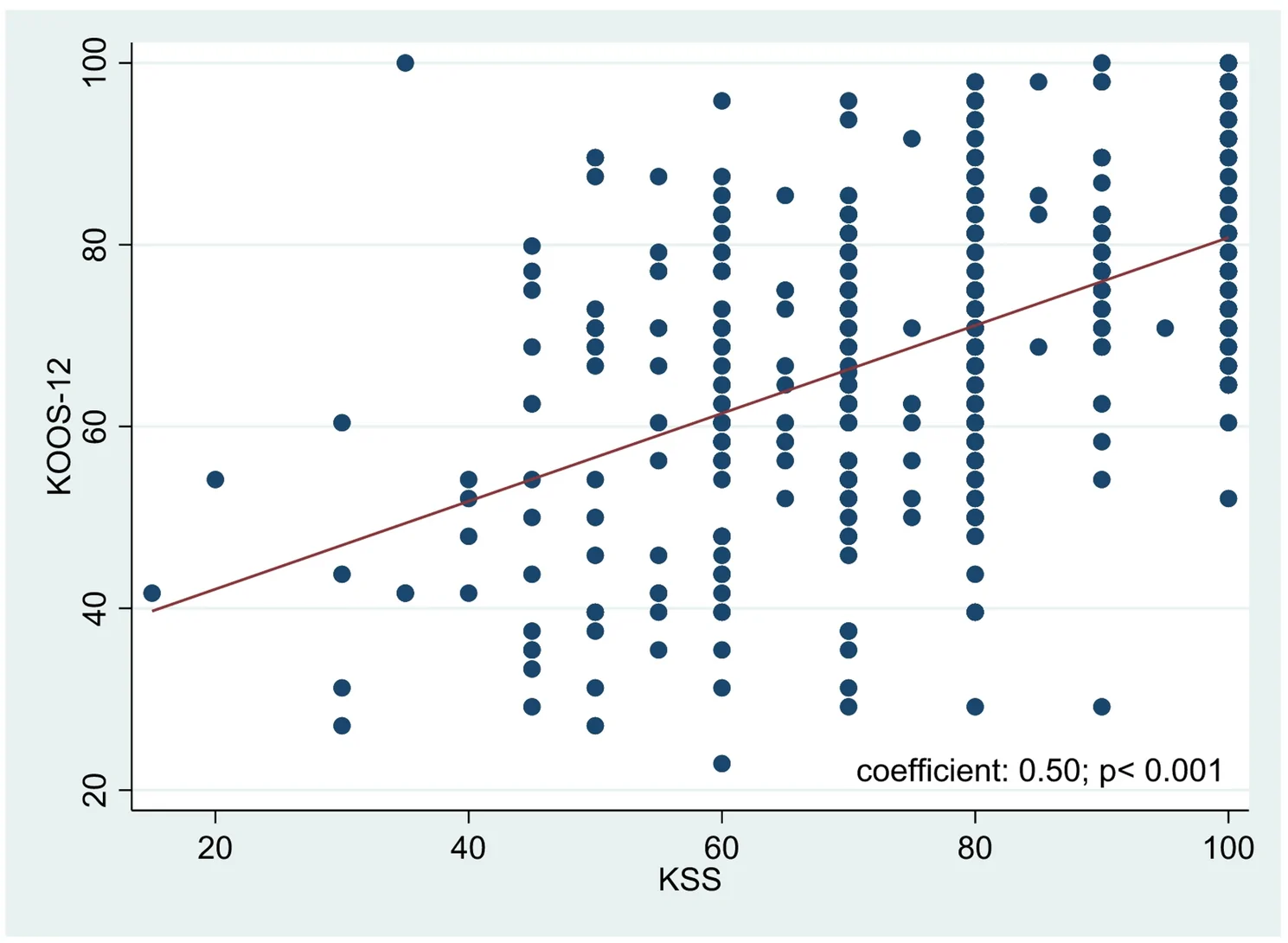
Correlation between KOOS-12 and KSS. Significant, positive and moderate correlation was observed between KOOS-12 score with KSS (coefficient: 0.50; p< 0.001)

**Fig. 3:**
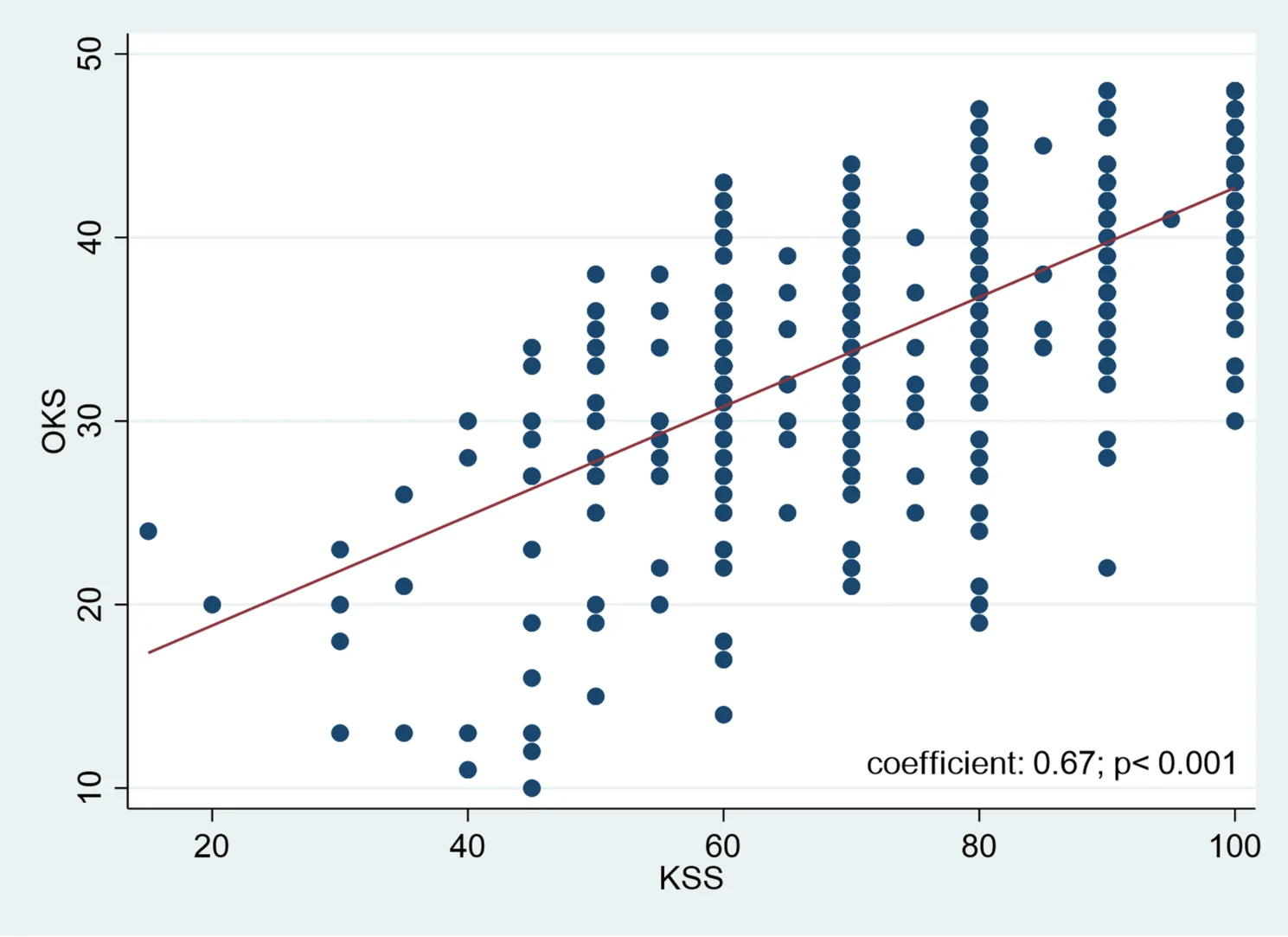
Correlation between KSS and OKS. Significant. positive and moderate correlation was observed between KOOS-12 score with KSS, (coefficient: 0.50; p < 0.001)

A significant strong positive correlation was found between KOOS-12 and OKS (coefficient: 0.79; p< 0.001), and a significant moderate positive correlation was found between the KOOS-12 and KSS (coefficient: 0.50; p< 0.001). A significant moderate positive correlation was found between the OKS and KSS (coefficient: 0.67; p< 0.001) (Table 3). Scatter plots with a best fit line for each pair of the measured PROMs (OSS, KSS, KOOS-12) are as depicted in Figs. 1, 2, 3 After adjustment for age and gender, 6 conversion equations were derived for the measured PROMs as summarized in Table 3.

### Internal validation

Overall, moderate to strong correlation was found between the actual and equation-derived (predicted) scores, ranging from the weakest strength observed in the conversion between KOOS-12 and KSS (coefficient: 0.53) and the strongest strength observed in the conversion between KOOS-12 and OKS (coefficient: 0.80). The mean difference between the equation-derived (predicted) and actual scores ranged from the smallest mean difference of -0.31 seen in the conversion of OKS to KSS (95% CI: -2.41, 1.80) to the largest mean difference of 1.68 seen in the conversion of OKS to KOOS-12 (95% CI: -0.18, 3.54). Within-individual difference between the equation-derived (predicted) and actual scores had great variation, ranging from the smallest difference of -12.4 to 12.7 (range: 25.1) in the conversion of KOOS-12 to OKS, to the largest difference of -35.9 to 44.4 (range: 80.3) seen in the conversion of KOOS-12 to KSS. Details of the internal validation of the derived conversion equations are displayed in Table 3.

**Table 1:** Demographic characteristics of the participants (*n* = 131)

Characteristics		Output
Age in years, mean ± SD		63.07 ± 7.84
Gender, n (%)	Male Female	44 (33.6) 87 (66.4)
Ethnicity, n (%)	Chinese Malay Indian Others	102 (77.9) 16 (12.2) 11 (8.4) 2 (1.5)
Employment status, n (%)	Employed Unemployed Homemaker Retired	77 (61.6) 7 (5.6) 12 (9.6) 29 (23.2)
BMI in kg/m^2^, mean ± SD		25.42 ± 6.73
Side of arthritis, n (%)	Left Right Both	33 (25.2) 46 (35.1) 52 (39.7)
KL grade, n (%)	2 3 4	23 (18.7) 56 (45.5) 44 (35.8)
Duration of osteoarthritis in years, median (IQR)		3 (1, 6)

Abbreviations: SD = Standard deviation; KL = Kellgren and Lawrence; BMI = Body Mass Index

**Table 2:** Summary of KOOS-12 OKS and KSS at different time points

Time point		KOOS	OKS	KSS
Baseline	N Mean ± SD Min - Max	131 66.58 ± 15.39 27.08–95.83	131 34.23 ± 7.30 13–47	131 75.50 ± 17.26 30–100
3 Months	N Mean ± SD Min - Max	114 65.99 ± 17.77 22.92–100.00	114 34.04 ± 7.70 12–48	114 71.97 ± 18.39 20–100
6 Months	N Mean ± SD Min - Max	88 68.33 ± 17.51 29.17–100.00	88 35.52 ± 8.04 10–48	88 74.03 ± 17.32 20–100
1 Year	N Mean ± SD Min - Max	90 72.06 ± 16.31 29.17–100.00	90 36.23 ± 7.35 13–48	90 75.67 ± 16.47 30–100
2 Year	N Mean ± SD Min - Max	75 70.00 ± 18.21 27.08–100.00	75 37.17 ± 7.47 15–48	74 75.07 ± 17.03 15–100

Abbreviations: SD = Standard deviation; IQR = Inter Quartile Range; KOOS = Knee Injury and Osteoarthritis Outcome Score; OKS = Oxford Knee Score; KSS = Knee Society Score

**Table 3:** Conversion equation and testing results

Conversion desired	Conversion Equation	Adjusted *R*^2^	Mean Difference between Actual and Predicted (95% CI)	Correlation between Actual and predicted	Within-Individual differences between actual and predicted range (min - max)
OKS → KOOS-12	KOOS-12 = -12.64 + (OKS x 1.70) + (Age x 0.33) + (0.77 if Female)	0.6374	1.68 (-0.18, 3.54)	0.80	-19.52 to 28.30
OKS → KSS	KSS = 46.73 + (OKS x 1.52) – (Age x 0.40) – (0.35 if Female)	0.4750	-0.31 (-2.41, 1.80)	0.69	-24.42 to 29.50
KOOS-12 → KSS	KSS = 72.79 + (KOOS-12 × 0.56) – (Age x 0.55) – (2.36 if Female)	0.3029	-0.77 (-1.69, 3.23)	0.53	-35.86 to 44.40
KOOS-12 → OKS	OKS = 17.06 + (KOOS-12 × 0.37) - Age x 0.10) - (1.33 if Female)	0.6297	0.77 (0.00, 1.55)	0.80	-12.43 to 12.69
KSS → OKS	OKS = 3.50 + (KSS x 0.30) + (Age x 0.16) - (1.36 if Female)	0.4739	0.36 (-0.50, 1.35)	0.68	-13.46 to 14.62
KSS → KOOS-12	KOOS-12 = -6.58 + (KSS x 0.51) + (Age x 0.60) – (1.55 if Female)	0.3160	-0.93 (-3.52, 1.67)	0.53	-25.70 to 31.97

Abbreviations: KOOS = Knee Injury and Osteoarthritis Outcome Score; OKS = Oxford Knee Score; KSS = Knee Society Score

Scoring range for: KOOS-12: 0-100; OKS: 0-48; KSS: 0-100

A significant positive, strong correlation was observed between KOOS-12 and OKS (coefficient: 0.79; *p* < 0.001).

## Discussion

Over the years, there has been a growing number of PROMs used to measure outcomes in KOA [16]. Significant heterogeneity exists among these instruments that measure similar constructs, making it difficult for comparison and pooling of outcomes between studies. Chamorro-Moriana et al. performed a systematic review of outcome measured used to assess knee function and found over 40 validated instruments measuring knee function [16]. Of these, the KOOS-12, OKS and KSS are three common instruments used to measure knee function and outcomes, including KOA [16, 17]. While all these instruments aim to measure knee function and outcomes, they vary in terms of their route of administration, measurement components and limitations. The KOOS-12 and OKS are both patient-reported questionnaires measuring knee pain and function, while the KSS requires both patient-reported outcomes such as their level of function as well as clinician input on objective findings such as knee alignment and range of motion [3–5]. 

To achieve greater data harmonization and comparison of studies with different PROMs, crosswalks of outcome measures have been previously evaluated in other orthopaedic conditions, which allow for estimation of PROMs measuring similar constructs allow studies or datasets with heterogenous outcome measures to be combined and compared, supporting more robust analyses and the exploration of new research questions [11, 18]. Nonetheless, similar crosswalks remain lacking in KOA, especially for non-operative population. While some studies have studied the correlation of PROMs in knee arthroplasty patients, crosswalks performed and validated in non-operatively treated knee OA is lacking. Soh et al. developed bi-directional crosswalk between OKS and KOOS-12 based on data from the Australian Orthopaedic Association National Joint Replacement Registry for patients undergoing knee arthroplasty. Similar to our study results, Soh et al. also found good correlation between OKS and KOOS-12 scores in their knee arthroplasty population [10]. Ackerman et al. performed a systematic review in 2024 of crosswalks performed for PROMs in shoulder, hip and knee surgery and found 4 crosswalk studies performed for OKS and EQ-5D-3 L, 1 crosswalk study performed for OKS and KOOS-joint replacement (KOOS-JR) and 1 study for OKS and KOOS-12 (Soh et al. as mentioned above) [8]. Crosswalks of outcome measures derived from arthroplasty populations may not be fully generalizable to non-operatively treated KOA or other knee conditions as operative patients are more likely to have significant pain and disability before operation (poor pre-op scores) coupled with significant symptom relieve post operatively (post op score), compared to the general non-operatively treated KOA population who are more likely to have a wider spectrum of the severity in terms of their symptoms and disability.

Our study findings have a few key clinical implications. In a climate where there exists significant outcome measure heterogeneity with a lack of any benchmark outcome measure in KOA as highlighted by multiple systematic review and meta-analysis in KOA, our study is a step towards providing greater data harmonization by using the conversion equations in future systematic review or meta-analysis [19, 20]. Our results show that the derived conversion equations provided a good estimate for conversion of the studied mean outcome scores (e.g. deriving the mean KOOS-12 from a mean OKS). Therefore, the conversion equations would allow consolidation and comparison of different outcome score (OKS, KSS, KOOS-12) across different studies in addressing novel questions with greater statistical power, as well as quality improvement projects which compare historical data and newer data measured with different outcome measures. Nonetheless, it is important to note that the conversion equations derived from our study should not be used to convert or predict outcome scores for each other on an individual level due to high within-individual variability. The high variability of outcome scores at an individual level found in our study on internal validation was not unique, with high within-individual variability also found in previous crosswalks of outcome measures used in knee and shoulder arthroplasty patients [11, 21]. 

### Strengths

To the authors’ knowledge, this was the first study which developed and validated bi-directional crosswalks for three different outcome measures (for OKS, KOOS-12 and KSS) in patients with non-operatively treated KOA.

## Study limitations

Limitations of this study include the fact that the study results may not be generalizable to other study populations. Our study included Asian patients with non-operatively treated KOA, which limits its generalizability to other populations such as operatively treated KOA populations or other knee conditions using similar outcome measures. Moreover, while the KSS, OKS, and KOOS-12 all assess overlapping measures (e.g., knee function), they remain differences in the construct of these instruments. Therefore, correlations or conversions between these instruments may still be limited by construct non-equivalence and are best interpreted at the group level rather than individual level. Lastly, although all available paired outcomes were included in the analyses, there were missing follow-up data which could introduce attrition bias and affect the robustness of the observed crosswalk equations.

## Conclusions

In our cohort of patients with non-operatively treated knee OA, moderate to strong correlations were found between the OKS, KOOS-12, and KSS. Six regression-based crosswalk equations were derived, which allow conversion between OKS, KOOS-12, and KSS scores. Internal validation showed moderate to good correlation between the equation-derived and actual mean outcome scores, although substantial variability was noted at the individual level. These equations may support pooling and comparison of studies using these heterogenous outcome measures, but should not be used for individual-level score estimations.

## Data Availability

Data are available upon request from the corresponding author, who can be contact through email, for clarification or collaboration for future studies.

## Abbreviations

KOA: Knee osteoarthritis
OKS: Oxford Knee Score
KOOS-12: Knee Injury and Osteoarthritis Outcome Score
KSS: Knee Society Score
PROMs: Patient-reported outcome measures
